# Supplementary material for: Stacking Model‐Based Classifiers for Dealing With Multiple Sets of Noisy Labels
Source: Biom J. 2025 Mar 12;67(2):e70042. doi: 10.1002/bimj.70042 (PMC11898607; doi:10.1002/bimj.70042)
Supplement: Supplementary file 1 — Supporting Information [file BIMJ-67-e70042-s001.zip › density-based-ensemble-model-main/competitors_real_data/netlab-master/netlab-master/help/index.htm]

NETLAB Reference Documentation


# NETLAB Online Reference Documentation

Welcome to the NETLAB online reference documentation.
The NETLAB simulation software is designed to provide all the tools necessary
for principled and theoretically well founded application development. The
NETLAB library is based on the approach and techniques described in *Neural
Networks for Pattern Recognition* (Bishop, 1995). The library includes software
implementations of a wide range of data analysis techniques, many of which are
not widely available, and are rarely, if ever, included in standard neural
network simulation packages.

The online reference documentation provides direct hypertext links to specific Netlab function descriptions.

If you have any comments or problems to report, please contact Ian Nabney (i.t.nabney@aston.ac.uk) or Christopher Bishop (c.m.bishop@aston.ac.uk).

# Index

An alphabetic list of functions in Netlab.

`conffig`: Display a confusion matrix. `confmat`: Compute a confusion matrix. `conjgrad`: Conjugate gradients optimization. `consist`: Check that arguments are consistent. `convertoldnet`: Convert pre-2.3 release MLP and MDN nets to new format `datread`: Read data from an ascii file. `datwrite`: Write data to ascii file. `dem2ddat`: Generates two dimensional data for demos. `demard`: Automatic relevance determination using the MLP. `demev1`: Demonstrate Bayesian regression for the MLP. `demev2`: Demonstrate Bayesian classification for the MLP. `demev3`: Demonstrate Bayesian regression for the RBF. `demgauss`: Demonstrate sampling from Gaussian distributions. `demglm1`: Demonstrate simple classification using a generalized linear model. `demglm2`: Demonstrate simple classification using a generalized linear model. `demgmm1`: Demonstrate density modelling with a Gaussian mixture model. `demgmm3`: Demonstrate density modelling with a Gaussian mixture model. `demgmm4`: Demonstrate density modelling with a Gaussian mixture model. `demgmm5`: Demonstrate density modelling with a PPCA mixture model. `demgp`: Demonstrate simple regression using a Gaussian Process. `demgpard`: Demonstrate ARD using a Gaussian Process. `demgpot`: Computes the gradient of the negative log likelihood for a mixture model. `demgtm1`: Demonstrate EM for GTM. `demgtm2`: Demonstrate GTM for visualisation. `demhint`: Demonstration of Hinton diagram for 2-layer feed-forward network. `demhmc1`: Demonstrate Hybrid Monte Carlo sampling on mixture of two Gaussians. `demhmc2`: Demonstrate Bayesian regression with Hybrid Monte Carlo sampling. `demhmc3`: Demonstrate Bayesian regression with Hybrid Monte Carlo sampling. `demkmean`: Demonstrate simple clustering model trained with K-means. `demknn1`: Demonstrate nearest neighbour classifier. `demmdn1`: Demonstrate fitting a multi-valued function using a Mixture Density Network. `demmet1`: Demonstrate Markov Chain Monte Carlo sampling on a Gaussian. `demmlp1`: Demonstrate simple regression using a multi-layer perceptron `demmlp2`: Demonstrate simple classification using a multi-layer perceptron `demnlab`: A front-end Graphical User Interface to the demos `demns1`: Demonstrate Neuroscale for visualisation. `demolgd1`: Demonstrate simple MLP optimisation with on-line gradient descent `demopt1`: Demonstrate different optimisers on Rosenbrock's function. `dempot`: Computes the negative log likelihood for a mixture model. `demprgp`: Demonstrate sampling from a Gaussian Process prior. `demprior`: Demonstrate sampling from a multi-parameter Gaussian prior. `demrbf1`: Demonstrate simple regression using a radial basis function network. `demsom1`: Demonstrate SOM for visualisation. `demtrain`: Demonstrate training of MLP network. `dist2`: Calculates squared distance between two sets of points. `eigdec`: Sorted eigendecomposition `errbayes`: Evaluate Bayesian error function for network. `evidence`: Re-estimate hyperparameters using evidence approximation. `fevbayes`: Evaluate Bayesian regularisation for network forward propagation. `gauss`: Evaluate a Gaussian distribution. `gbayes`: Evaluate gradient of Bayesian error function for network. `glm`: Create a generalized linear model. `glmderiv`: Evaluate derivatives of GLM outputs with respect to weights. `glmerr`: Evaluate error function for generalized linear model. `glmevfwd`: Forward propagation with evidence for GLM `glmfwd`: Forward propagation through generalized linear model. `glmgrad`: Evaluate gradient of error function for generalized linear model. `glmhess`: Evaluate the Hessian matrix for a generalised linear model. `glminit`: Initialise the weights in a generalized linear model. `glmpak`: Combines weights and biases into one weights vector. `glmtrain`: Specialised training of generalized linear model `glmunpak`: Separates weights vector into weight and bias matrices. `gmm`: Creates a Gaussian mixture model with specified architecture. `gmmactiv`: Computes the activations of a Gaussian mixture model. `gmmem`: EM algorithm for Gaussian mixture model. `gmminit`: Initialises Gaussian mixture model from data `gmmpak`: Combines all the parameters in a Gaussian mixture model into one vector. `gmmpost`: Computes the class posterior probabilities of a Gaussian mixture model. `gmmprob`: Computes the data probability for a Gaussian mixture model. `gmmsamp`: Sample from a Gaussian mixture distribution. `gmmunpak`: Separates a vector of Gaussian mixture model parameters into its components. `gp`: Create a Gaussian Process. `gpcovar`: Calculate the covariance for a Gaussian Process. `gpcovarf`: Calculate the covariance function for a Gaussian Process. `gpcovarp`: Calculate the prior covariance for a Gaussian Process. `gperr`: Evaluate error function for Gaussian Process. `gpfwd`: Forward propagation through Gaussian Process. `gpgrad`: Evaluate error gradient for Gaussian Process. `gpinit`: Initialise Gaussian Process model. `gppak`: Combines GP hyperparameters into one vector. `gpunpak`: Separates hyperparameter vector into components. `gradchek`: Checks a user-defined gradient function using finite differences. `graddesc`: Gradient descent optimization. `gsamp`: Sample from a Gaussian distribution. `gtm`: Create a Generative Topographic Map. `gtmem`: EM algorithm for Generative Topographic Mapping. `gtmfwd`: Forward propagation through GTM. `gtminit`: Initialise the weights and latent sample in a GTM. `gtmlmean`: Mean responsibility for data in a GTM. `gtmlmode`: Mode responsibility for data in a GTM. `gtmmag`: Magnification factors for a GTM `gtmpost`: Latent space responsibility for data in a GTM. `gtmprob`: Probability for data under a GTM. `hbayes`: Evaluate Hessian of Bayesian error function for network. `hesschek`: Use central differences to confirm correct evaluation of Hessian matrix. `hintmat`: Evaluates the coordinates of the patches for a Hinton diagram. `hinton`: Plot Hinton diagram for a weight matrix. `histp`: Histogram estimate of 1-dimensional probability distribution. `hmc`: Hybrid Monte Carlo sampling. `kmeans`: Trains a k means cluster model. `knn`: Creates a K-nearest-neighbour classifier. `knnfwd`: Forward propagation through a K-nearest-neighbour classifier. `linef`: Calculate function value along a line. `linemin`: One dimensional minimization. `maxitmess`: Create a standard error message when training reaches max. iterations. `mdn`: Creates a Mixture Density Network with specified architecture. `mdn2gmm`: Converts an MDN mixture data structure to array of GMMs. `mdndist2`: Calculates squared distance between centres of Gaussian kernels and data `mdnerr`: Evaluate error function for Mixture Density Network. `mdnfwd`: Forward propagation through Mixture Density Network. `mdngrad`: Evaluate gradient of error function for Mixture Density Network. `mdninit`: Initialise the weights in a Mixture Density Network. `mdnpak`: Combines weights and biases into one weights vector. `mdnpost`: Computes the posterior probability for each MDN mixture component. `mdnprob`: Computes the data probability likelihood for an MDN mixture structure. `mdnunpak`: Separates weights vector into weight and bias matrices. `metrop`: Markov Chain Monte Carlo sampling with Metropolis algorithm. `minbrack`: Bracket a minimum of a function of one variable. `mlp`: Create a 2-layer feedforward network. `mlpbkp`: Backpropagate gradient of error function for 2-layer network. `mlpderiv`: Evaluate derivatives of network outputs with respect to weights. `mlperr`: Evaluate error function for 2-layer network. `mlpevfwd`: Forward propagation with evidence for MLP `mlpfwd`: Forward propagation through 2-layer network. `mlpgrad`: Evaluate gradient of error function for 2-layer network. `mlphdotv`: Evaluate the product of the data Hessian with a vector. `mlphess`: Evaluate the Hessian matrix for a multi-layer perceptron network. `mlphint`: Plot Hinton diagram for 2-layer feed-forward network. `mlpinit`: Initialise the weights in a 2-layer feedforward network. `mlppak`: Combines weights and biases into one weights vector. `mlpprior`: Create Gaussian prior for mlp. `mlptrain`: Utility to train an MLP network for demtrain `mlpunpak`: Separates weights vector into weight and bias matrices. `netderiv`: Evaluate derivatives of network outputs by weights generically. `neterr`: Evaluate network error function for generic optimizers `netevfwd`: Generic forward propagation with evidence for network `netgrad`: Evaluate network error gradient for generic optimizers `nethess`: Evaluate network Hessian `netinit`: Initialise the weights in a network. `netopt`: Optimize the weights in a network model. `netpak`: Combines weights and biases into one weights vector. `netunpak`: Separates weights vector into weight and bias matrices. `olgd`: On-line gradient descent optimization. `pca`: Principal Components Analysis `plotmat`: Display a matrix. `ppca`: Probabilistic Principal Components Analysis `quasinew`: Quasi-Newton optimization. `rbf`: Creates an RBF network with specified architecture `rbfbkp`: Backpropagate gradient of error function for RBF network. `rbfderiv`: Evaluate derivatives of RBF network outputs with respect to weights. `rbferr`: Evaluate error function for RBF network. `rbfevfwd`: Forward propagation with evidence for RBF `rbffwd`: Forward propagation through RBF network with linear outputs. `rbfgrad`: Evaluate gradient of error function for RBF network. `rbfhess`: Evaluate the Hessian matrix for RBF network. `rbfjacob`: Evaluate derivatives of RBF network outputs with respect to inputs. `rbfpak`: Combines all the parameters in an RBF network into one weights vector. `rbfprior`: Create Gaussian prior and output layer mask for RBF. `rbfsetbf`: Set basis functions of RBF from data. `rbfsetfw`: Set basis function widths of RBF. `rbftrain`: Two stage training of RBF network. `rbfunpak`: Separates a vector of RBF weights into its components. `rosegrad`: Calculate gradient of Rosenbrock's function. `rosen`: Calculate Rosenbrock's function. `scg`: Scaled conjugate gradient optimization. `som`: Creates a Self-Organising Map. `somfwd`: Forward propagation through a Self-Organising Map. `sompak`: Combines node weights into one weights matrix. `somtrain`: Kohonen training algorithm for SOM. `somunpak`: Replaces node weights in SOM.

---

Copyright (c) Christopher M Bishop, Ian T Nabney (1996, 1997)
